# Biomarker screening for antenatal depression in women who underwent caesarean section: a matched observational study with plasma Lipidomics

**DOI:** 10.1186/s12888-019-2241-1

**Published:** 2019-08-27

**Authors:** Zhuoxi Wu, Peng Zhao, Zhonghong Long, Jie Li, Guiying Yang, Qingling Zhang, Guangyou Duan, Hong Li

**Affiliations:** 1 Department of Anesthesiology, Second Affiliated Hospital of Army Medical University, PLA, Chongqing, China; 2 Department of Psychology, Second Affiliated Hospital of Army Medical University, PLA, Chongqing, China

**Keywords:** Biomarker, Antenatal depression, Women undergoing caesarean section

## Abstract

**Background:**

Antenatal depression is a prevalent mental disorder in women who have undergone caesarean section, and it often presages adverse postoperative outcomes. Because of the lack of a laboratory-based diagnostic strategy, antenatal depression is mainly determined by a psychologist’s subjective judgment based on a structured clinical interview for established diagnostic criteria. However, the diagnostic accuracy rate for depression by non-psychiatrists is relatively low. Thus, this study aimed to use lipidomics to identify potential biomarkers related to antenatal depression in women who have undergone caesarean section.

**Methods:**

The study was designed as a matched prospective observational study. Singleton pregnant women scheduled to receive elective caesarean section, were screened for eligibility. Women diagnosed with major antenatal depression were matched with non-antenatal depression controls in terms of age (±1 year) and BMI (±1 kg/m^2^), and blood samples of the included matched pairs were collected. Subsequently, lipidomics of the plasma samples were performed using Ultra Performance Liquid Chromatography-mass spectrometry analysis to explore the differentially expressed lipids in women with or without antenatal depression.

**Results:**

In total, 484 pregnant women were screened; 66 subjects were recruited, including 33 subjects with major antenatal depression and 33 matched controls without antenatal depression. Thirty-five differentially expressed lipid metabolites were identified (*P* < 0.05). The area under the receiver operating characteristic curve of these lipid metabolites was 0.7 or larger; the area under curve for cholesterol sulfate was 0.823 (95% CI: 0.716–0.930), and that of PC (18:2 (2E, 4E)/0:0) was 0.778 (95%CI: 0.662–0.895). In the conditional logistic stepwise regression analysis, cholesterol sulfate (*P* = 0.009) and PC (18:2 (2E, 4E)/0:0) (*P* = 0.035) were also identified as effective predictive risk factors for antenatal depression.

**Conclusions:**

Women who had undergone caesarean section and experienced antenatal depression presented a significantly differentially expressed profile of plasma lipidomics compared to those who did not experience antenatal depression. Cholesterol sulfate and PC (18:2 (2E, 4E)/0:0) may be effective and specific lipidic biomarkers for the prediction of antenatal depression.

**Trial registration:**

China Clinical Trial Registration Center registration number: ChiCTR1800016230; date of registration: 21/05/2018.

## Background

Antenatal depression (AD) is a prevalent mental disorder that affects approximately 5–15% of pregnant women [1, 2], and as reported in one previous study, the incidence even reached 26–28% in China [2, 3]. Caesarean section is one of the most commonly used ways of maternal delivery, and the global caesarean section rate was 27.3% in 2008 according to a WHO survey [4]. Recent data showed that the caesarean section rate in China reached 34.9% in 2014 [5], while in the US in 2014, the rate was 32.2% [6]. It is estimated that tens of millions of women give birth through caesarean sections every year. Caesarean sections in women are often correlated with different psychological and socioeconomic conditions and involve different clinical complications compared to vaginal delivery. Previous studies have reported that the incidence of perinatal depression in women who have undergone caesarean sections is even higher than that in women who have had vaginal deliveries [7–10]. It is known that depressive disorders are associated with more severe postoperative pain, worse postoperative recovery, and long-term postpartum outcomes. In addition, women with untreated AD have more than seven times the risk of postpartum depression (PPD) than women without AD [11]. Therefore, preoperative identification of AD can be helpful for potential treatment to improve maternal outcomes in women who have undergone caesarean section. Additionally, it is important to consider AD before a caesarean section.

Due to the lack of a laboratory diagnostic strategy [12], identification of AD often requires a comprehensive clinical interview by a psychiatrist [13], involving a subjective judgment based on the clinical symptoms, behaviour, and psychological characteristics, etc. At present, the 10-item Edinburgh postpartum depression (EPDS-10) scales [14–16] are commonly used in AD screening and the diagnostic criteria mainly depend on the American Psychiatric Association’s Diagnostic and Statistical Manual of Mental Disorders, fifth edition (DSM-V) [17]. However, it is difficult for non-psychiatric doctors such as obstetricians and anaesthesiologists to accurately diagnose depression without the necessary long-term professional training [18, 19]. A credible meta-analysis [20] showed that the rate of false positives would surpass that of true positives by about 50% in primary care practices if the diagnosis of depression were performed by general practitioners (non-psychiatrists). One study indicated that diagnosis of AD is difficult because the physiological signs of pregnancy overlap with the symptoms of AD [21]. In addition, not all antenatal-depressed individuals voluntarily and willingly manifest emotional symptoms. Furthermore, in clinical practice, obstetricians and anaesthesiologists often pay little attention to AD before a caesarean delivery. Therefore, it is necessary to find an objective, fast, and practical indicator to help clinicians identify or predict AD in their daily clinical practices.

A ‘biomarker’ is defined as a specific biomolecule that is objectively measured and evaluated as an indicator of normal biological and pathogenic processes or pharmacological responses to a therapeutic intervention [22]. The National Institute of Mental Health (NIMH) Research Domain Criteria (RDoC) has set the discovery of biomarkers as a priority for clinical research [23]. Objective biomarkers that can be measured externally have been demonstrated to be good predictors of the personalized diagnosis and treatment of depression [22, 24]. For this purpose, metabolomics is currently a viable and widely used method for finding biomarkers for neuropsychological diseases [25]. Additionally, lipidomics are recommended and have been used in several previous studies to explore the potential biomarkers of depression disorders [26, 27]. However, at present, studies using lipidomics to explore biomarkers of AD are lacking. Therefore, this study aimed to use lipidomics to find potential biomarkers related to AD in women who had undergone caesarean section.

## Methods

### Ethical statement

The study protocol was approved by the Medical Ethics Committee of the Second Affiliated Hospital, Army Military Medical University (Approved ID: 2018-Research No. 033–01). The study was registered at the China Clinical Trial Registration Center (http://www. Chictr.org.cn/index.aspx) with registration number: ChiCTR1800016230. Prior to the study, written informed consent was provided by all subjects.

### Patients

The study was conducted in the Second Affiliated Hospital of the Army Military Medical University from May 2018 to August 2018. One day before the surgery, singleton pregnant women scheduled to receive elective caesarean section were screened for eligibility in the obstetric wards. The inclusion criteria were as follows: 1) patient age between 20 to 40 years; 2) American Society of Anesthesiologists (ASA) classification I ~ II; and 3) a full-term pregnancy. The exclusion criteria were as follows: 1) known history of mental disorders before pregnancy; 2) history of psychotropic drug abuse; 3) drug, alcohol or opioid abuse; 4) severe systemic diseases such as those concerning the heart, brain, liver, kidney and hematopoietic system; and 5) inability to cooperate for any reason.

### Study procedure

#### Sample size

The study was designed as a matched prospective observational study. The primary aim was to perform lipidomics analysis, which required the sample size of each group to be 30. To avoid the possible failure of the blood sample analysis due to various reasons, an additional 10% of the subjects were included. Thus, 33 subjects with AD and normal controls were included in the study.

#### AD diagnosis

The diagnosis of AD was performed based on a well-established method as described in a previous study [11]. First, an initial screening of depression was performed using the EPDS-10 which was delivered to women subjects. Thereafter, according to the DSM-V diagnostic criteria the severity of AD, whether moderate or major was determined by a professional psychiatrist. The screening and diagnosis process of the non-antenatal depression (NAD) group subjects who met the matching criteria, was ensured to be completely consistent with that of the antenatal depression (AD) group. Subjects with an EPDS-10 ≥ 10 [28] or if the subjects selected the answers B, C or D, for the 10th question and met the DSM-V major depression criteria were defined as the AD subjects. On the other hand, the NAD subjects included those who selected the answer A for the 10th item of the EPDS-10, EPDS-10 < 10 [28] and did not meet the DSM-V major depression criteria.

#### Screening and matching

**Fig. 1:**
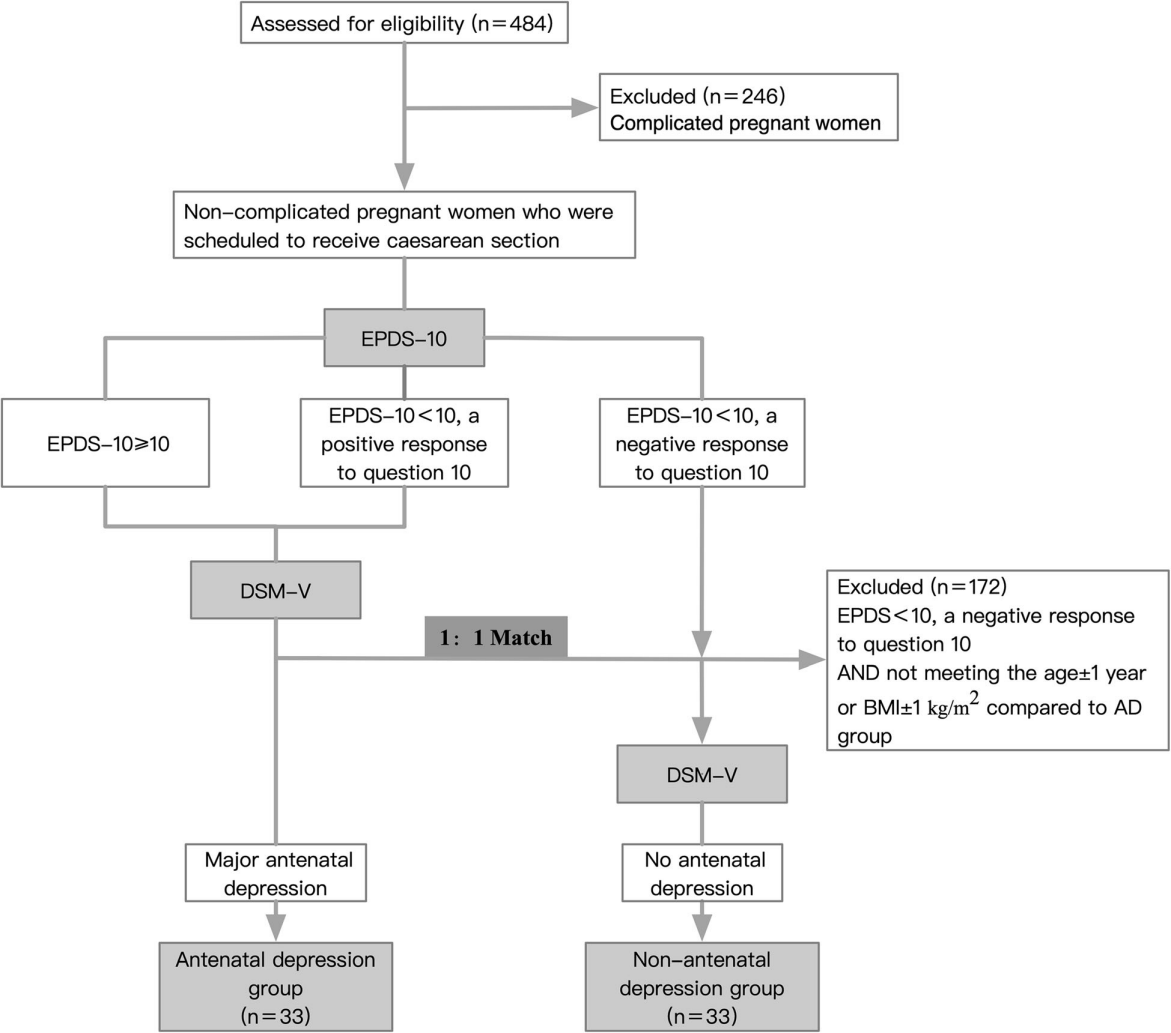
Flow diagram of inclusion criteria. DSM-V, Diagnostic and Statistical Manual of Mental Disorders, fifth edition; EPDS-10, 10-item Edinburgh Postnatal Depression Scale

Screening and matching were conducted according to a one-way process (Fig. 1). After one eligible positive subject was enrolled in the AD group, one NAD subject was selected, matched to the subject with AD in terms of age ± 1 year and BMI ±1 kg/m^2^. In subjects with an EPDS-10 ≥ 10 or a positive answer to the 10th item, the DSM-V diagnosis was performed by a psychiatric expert, subsequent to which the eligible positive subject was allocated to the AD group. In subjects with an EPDS-10 < 10 and with a negative answer to the 10th item, the DSM-V diagnosis was performed only if the subject met the matched criteria (age ± 1 year and BMI ±1 kg/m^2^) compared to the previously included subjects in the AD group. If both the EPDS and DSM-V had negative answers, the subjects were allocated to the Non-AD group. Cases with unsuccessful matches were considered invalid and were not used for matching in subsequent cases. The inclusion of subjects was terminated after the number of eligible matched subjects in both the AD group and the NAD group reached 33. Peripheral blood samples were collected only from the patients who were finally included in the study.

### Sample collection and preparation

After the completion of psychological tests, for inclusion of eligible women who met the matching criteria, 4–5 ml blood was collected from the elbow vein of each subject, using a heparin anticoagulation tube. Then, the blood sample was centrifuged for 5 min (4000×g, 4 °C), and aliquots (400 μL) of the plasma samples were stored in a − 80 °C freezer for ultra- performance liquid chromatography-mass spectrometry (UPLC-MS) analysis. Thereafter, 1 ml of methanol was added to a 300 μL aliquot of the sample and the resultant mixture was vortexed for 15 s, followed by the addition of 1 mL of chloroform and vortexing for another 5 min. The sample was centrifuged for 10 min at 12,000 rpm at 4 °C. The supernatant was then transferred to a new tube, and 300 μL of H_2_O were added, followed by vortexing for 5 min and centrifugation for 10 min at 12,000 rpm at 4 °C. The 200 μL lower phase was dried in a vacuum concentrator and stored at − 20 °C.

### Liquid chromatography-mass spectrometry conditions

A Thermo Scientific™ Q Exactive™ hybrid quadruple Orbitrap mass spectrometer (Thermo Fisher Scientific, Waltham, MA, USA) equipped with a HESI-II probe was employed. The positive and negative HESI-II spray voltages were 3.7 kV and 3.5 kV, respectively, the heated capillary temperature was 320 °C, the sheath gas pressure was 30 psi, the auxiliary gas setting was 10 psi, and the heated vaporizer temperature was 300 °C. Both the sheath gas and the auxiliary gas were nitrogen. The collision gas was also nitrogen at a pressure of 1.5 mTorr. The parameters of the full mass scan were as follows: a resolution of 70,000, an auto gain control target under 1 × 10^6^, a maximum isolation time of 50 ms, and an m/z range of 150–1500. The parameters of the dd-MS2 scan were as follows: a resolution of 17,500, an auto gain control target under 1 × 10^5^, a maximum isolation time of 50 ms, a loop count of the top 10 peaks, an isolation window of m/z 2, a normalized collision energy of 30v and an intensity threshold under 1 × 10^5^. The LC-MS system was controlled using Xcalibur 2.2 SP1.48 software (Thermo Fisher Scientific), and the data were collected and processed with the same software.

### Non-targeted lipidomics data processing

The data derived from Progenesis QI was imported into MetaboAnalyst 3.0 for multivariate statistical analysis. First, principal component analysis (PCA) was used to analyse the quality control (QC) samples and the other experimental samples, as well as to ensure uniform distribution among the samples and stability of the entire analysis process. Then, the partial least squares method (PLS-DA) analysis was used to distinguish between the overall difference in metabolic profiles of the AD and NAD groups. Meanwhile, the Variable Importance for the Projection (VIP) index was used to evaluate the influence of intensity and the explanatory ability of each metabolite expression pattern on the classification and discrimination of each group of samples, so as to assist in the screening of significant metabolites. Then, we adjusted the *P*-value of the T-test using the Benjamini–Hochberg procedure with the critical false discovery rate (FDR) set at 0.05. The reference standard for screening is VIP > 1 and an FDR <0.05. Bonferroni correction with *P* values multiplied 35 times are also presented in Table 2. The fold change was obtained by dividing the average of the AD group by that of the NAD group in the multiple tests. The two sets of data were analysed by a *t*-test that was used to generate a hot plot.

### Statistical analysis

Variables are presented as mean ± standard deviation (SD), number (percentage) or median with their respective interquartile ranges (IQR). The general demographic and clinical data between the AD and NAD groups were analysed using the Independent-Samples T Test, Chi-square test, or the Mann-Whitney U test. Receiver operating characteristic curve (ROC) analysis was performed for all of the identified molecular markers with statistical significance, and the value of the area under the ROC curve (AUC) was calculated. Conditional logistic regression analysis for AD was also performed. The total number of included cases in this study was 66. Since the number of subjects must be at least 10 times the number of included independent variables, as per the sample size inclusion principle for logistic regression analysis, 6 factors were considered in the current regression model. Thus, we chose the top six identified biomarkers with the maximum AUC as independent variables.

Then, an exploratory multiple regression analysis using the entire model was performed to investigate whether the main identified biomarker was affected by the clinical characteristics; age, BMI and EPDS; educational level (9 to 12 years = 1, over 12 years = 2), marital relationship (Bad = 1, General = 2, Good = 3) and sleep quality (Very poor – poor = 1, General = 2, Good -very good = 3) were regarded as independent variables of the main identified biomarker. All statistical analyses were performed via SPSS software version 24. A *P*-value of < 0.05 was considered statistically significant.

## Results

### General results

A total of 484 pregnant women were screened in the study; 246 cases who presented with complications were excluded, and 172 cases were excluded because their age and BMI did not match the criteria for inclusion in the AD group, according to the study design. Finally, 66 subjects were included for analysis comprising 33 subjects with AD and 33 without AD. The general demographic and clinical data of the subjects are presented in Table 1. There were no statistical differences with respect to age, BMI, gestational weeks, marital relationship, and education level between the two groups. The sleep quality in the AD group was worse than that in the NAD group (*P* = 0.011).

**Table 1:** Demographic and clinical characteristics of the antenatal depression and non-antenatal depression subjects

Characteristics	Antenatal depression (*n* = 33)	Non-antenatal depression (*n* = 33)	*P*-value
Age (year)^a^	30.7 ± 3.6	31.0 ± 3.6	0.760
Range	23–39	23–40	–
BMI (kg/m^2^) ^a^	27.6 ± 2.6	27.5 ± 2.5	0.906
Gestational weeks ^a^	37.6 ± 1.4	38.2 ± 1.3	0.096
Marital relationship^c^			0.109
Bad	0 (0%)	0 (0%)	
General	6 (18.2%)	1 (3.0%)	
good	27 (81.8%)	32 (97.0%)	
Educational level (year)^c^			0.614
9 to 12 years	14 (42.4%)	12 (36.4%)	
≥12 years	19 (57.6%)	21 (63.6%)	
Sleep quality^c^			0.011
Very poor-poor	10 (30.3%)	1 (3.0%)	
General	15 (45.5%)	19 (57.6%)	
Good-very good	8 (24.2%)	13 (39.4%)	

Data are described as mean ± standard deviation, median with interquartile range or number (percentage)

^a^Analysed by the independent-samples t-test

^b^Analysed by the Chi-square test

^c^Analysed by the Mann-Whitney U test

### Metabonomic analysis of plasma obtained from AD and NAD subjects

**Fig. 2:**
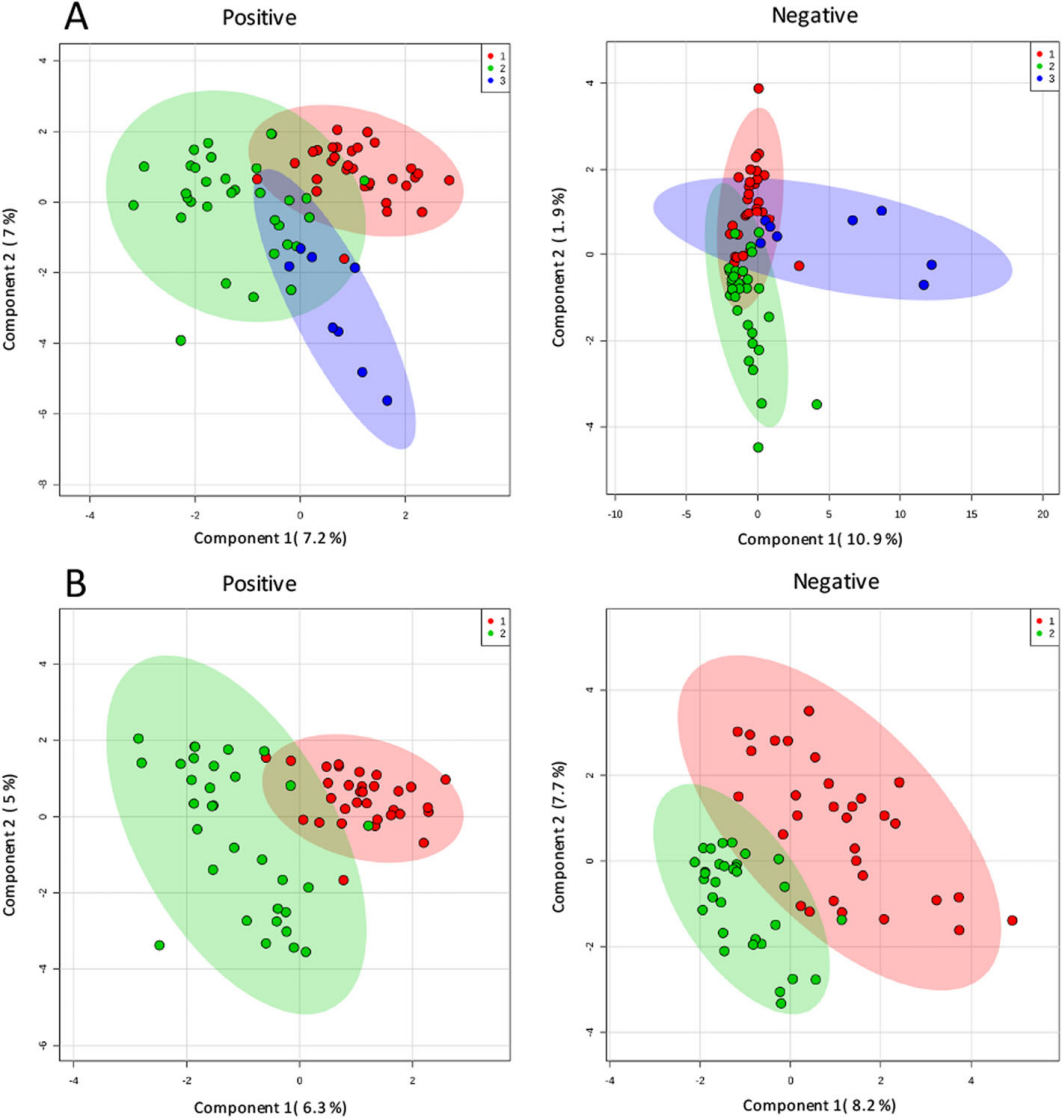
**a** Principal component analysis score scatter plots in a positive model and in a negative model. **b** Partial least square discriminant analysis score scatter plots of antenatal depression versus non-antenatal depression in a positive model and in a negative model. Red circle = non-antenatal depression group, green circle = antenatal depression group, blue circle = quality control samples

**Fig. 3:**
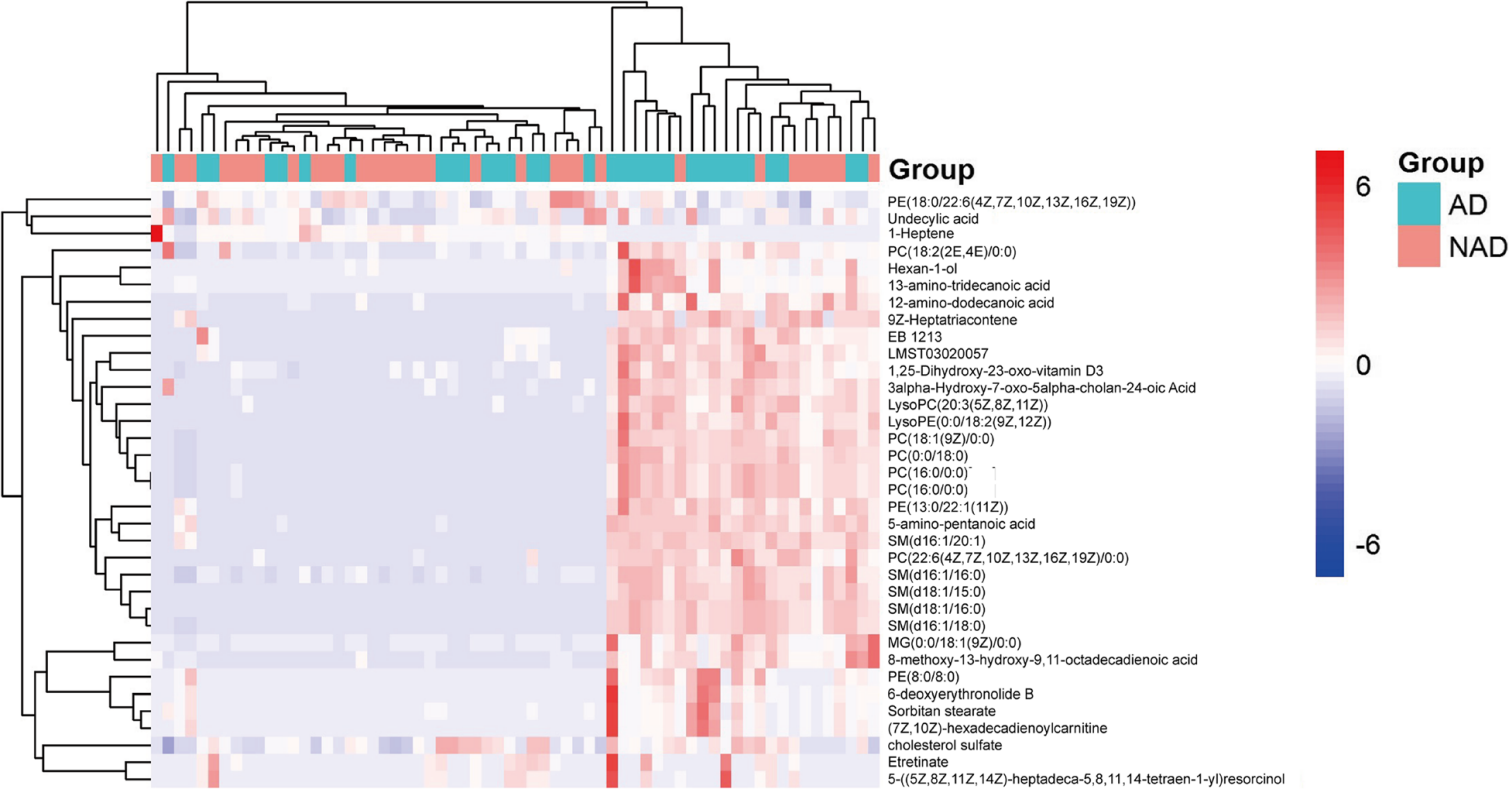
Heat map of the differentially expressed lipid metabolites in women with antenatal depression versus those without antenatal depression. AD, antenatal depression; NAD, non-antenatal depression

A total of 33 pairs of plasma samples were analysed using lipidomics. The PCA score plot for all the samples is shown in Fig. 2a. The relatively clustered QC samples show that the system is reproducible and that the method is stable and reliable. The PLS-DA score map for the positive- and negative-mode (Fig. 2b) showed that the two groups were significantly different. Using a combination of multidimensional and single-dimensional analyses, we identified 35 metabolites with significant differences between the NAD and AD groups (*P* < 0.05, VIP>1). The details of these differentially expressed metabolites are shown in Table 2. The heat map of the differentially expressed metabolites of the NAD and AD groups is presented in Fig. 3; it is evident that the expression of the identified lipids in the AD group is significantly higher than in the NAD group.

**Table 2:** Identified differential metabolites between the antenatal depression and non-antenatal depression groups

Metabolites	AD vs NAD					
	ESI^+/−^	FC	FDR	VIP	*P*-value	*Bonferroni corrected*
SM (d18:1/16:0)	+	1.88	< 0.001	5.07	< 0.001	< 0.001
SM (d16:1/18:0)	+	2.27	< 0.001	5.42	< 0.001	< 0.001
9Z-Heptatriacontene	+	2.03	< 0.001	8.09	< 0.001	< 0.001
PC (16:0/0:0)	–	2.00	< 0.001	2.20	< 0.001	< 0.001
EB 1213	+	3.30	< 0.001	3.83	< 0.001	< 0.001
3alpha-Hydroxy-7-oxo-5alpha-cholan-24-oic Acid	+	2.55	0.001	3.72	0.001	0.035
PC (22:6(4Z,7Z,10Z,13Z,16Z,19Z)/0:0)	+	2.65	0.001	1.38	0.002	0.07
cholesterol sulfate	–	2.49	0.001	1.46	0.003	0.105
PC (18:2(2E,4E)/0:0)	+	2.70	0.001	1.12	0.003	0.105
LMST03020057	+	2.48	0.001	3.84	0.004	0.14
PC(16:0/0:0)	–	2.07	0.001	1.57	0.004	0.14
PC(0:0/18:0)	–	2.03	0.027	1.58	0.004	0.14
Hexan-1-ol	+	3.61	0.001	2.83	0.006	0.21
PE(13:0/22:1(11Z))	+	2.34	0.001	1.16	0.006	0.21
LysoPE (0:0/18:2(9Z,12Z))	+	2.29	0.001	1.01	0.006	0.21
1,25-Dihydroxy-23-oxo-vitamin D3	+	2.04	0.001	1.72	0.007	0.245
SM(d16:1/16:0)	–	2.36	0.001	1.35	0.007	0.245
Undecylic acid	+	2.44	0.001	0.97	0.008	0.28
SM(d18:1/15:0)	+	2.18	0.001	1.55	0.009	0.315
5-amino-pentanoic acid	+	2.01	0.001	1.03	0.009	0.315
Sorbitan stearate	+	4.45	0.001	1.46	0.009	0.315
SM(d16:1/20:1)	–	2.06	0.001	1.20	0.009	0.315
PC(18:1(9Z)/0:0)	+	2.29	0.001	3.48	0.010	0.35
LysoPC (20:3(5Z,8Z,11Z))	+	2.25	0.001	1.56	0.010	0.35
PE(8:0/8:0)	+	3.16	0.001	1.83	0.010	0.35
Etretinate	–	3.34	0.001	1.21	0.011	0.315
13-amino-tridecanoic acid	+	3.08	0.001	3.78	0.011	0.315
6-deoxyerythronolide B	+	4.77	0.001	2.60	0.013	0.455
PE(18:0/22:6(4Z,7Z,10Z,13Z,16Z,19Z))	–	2.07	0.002	1.72	0.015	0.525
(7Z,10Z)-hexadecadienoylcarnitine	+	3.64	0.002	1.53	0.016	0.56
12-amino-dodecanoic acid	+	2.47	0.002	3.66	0.017	0.595
5-((5Z,8Z,11Z,14Z)-heptadeca-5,8,11,14-tetraen-1-yl)resorcinol	–	4.58	0.002	1.33	0.017	0.595
MG(0:0/18:1(9Z)/0:0)	+	2.13	0.002	7.62	0.027	0.945
1-Heptene	+	0.47	0.003	3.77	0.034	1.19
8-methoxy-13-hydroxy-9,11-octadecadienoic acid	+	2.03	0.003	1.47	0.034	1.19

*AD* antenatal depression, *NAD* non-antenatal depression, *ESI* electrospray ionization, *FC* fold change, *FDR* false discovery rate, *VIP* variable importance in the projection

### Identification of AD biomarkers

**Fig. 4:**
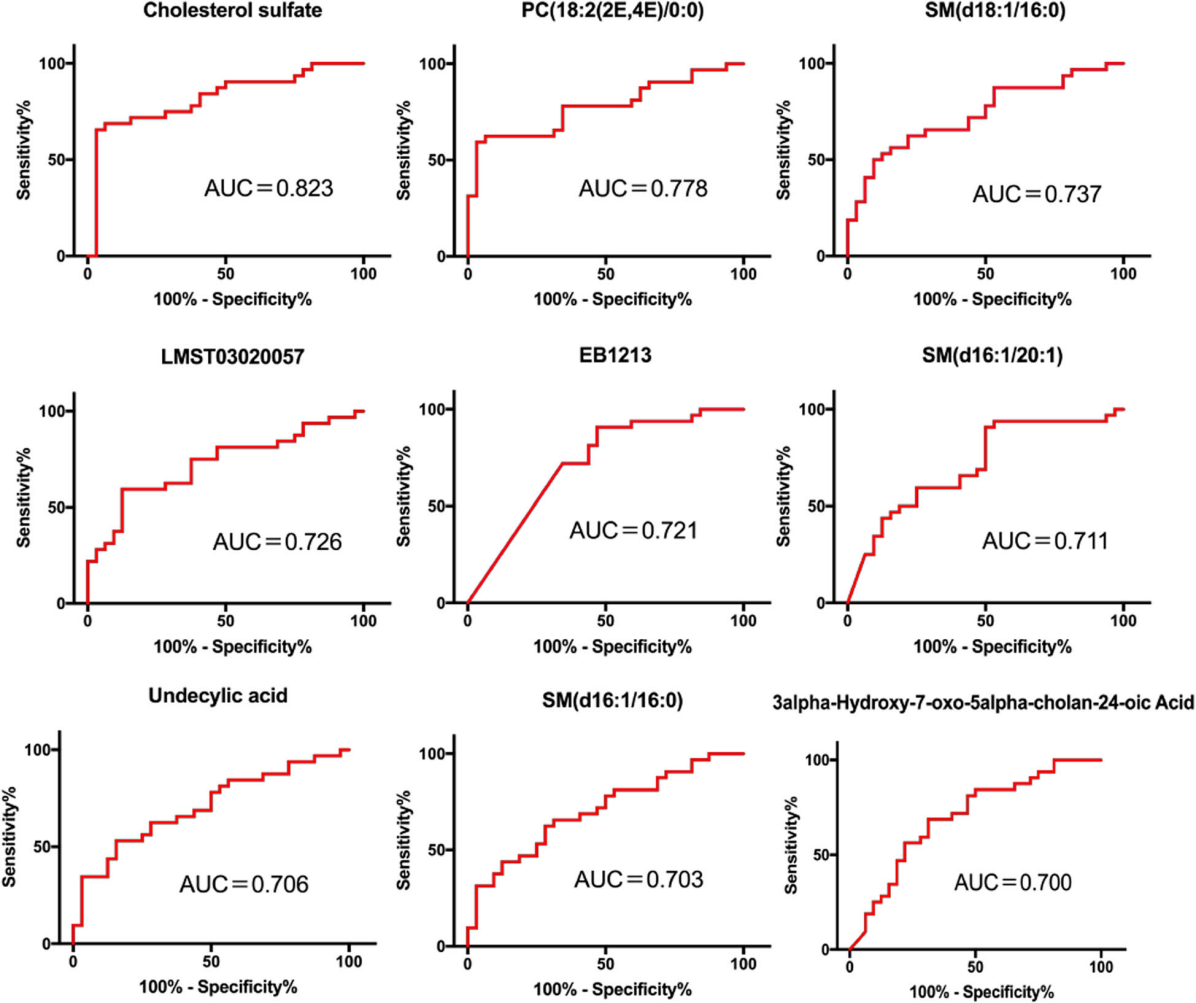
ROC curves of lipid metabolites. The area under the curve is equal to or greater than 0.7 for antenatal depression and non-antenatal depression diagnosis. AUC, area under the ROC curve

We performed ROC analysis for 35 differential lipids to determine their diagnostic performance. The differential lipids with AUC area > 0.7 are shown in Fig. 4. The AUC of cholesterol sulfate (CS) was 0.823 (95% CI: 0.716–0.930), according to the Youden index, the sensitivity of the best cut-off threshold (16,340.3052) was 0.969, and the specificity was 0.656. The accuracy, false positive rate, and false negative rate of CS were 0.813, 0.343, and 0.031, respectively. In addition, the AUC of PC (18:2 (2E, 4E)/0:0) was 0.778 (95% CI: 0.662–0.895), SM (d18:1/16:0) was 0.737 (95% CI: 0.615–0.860), LMST03020057 was 0.727 ((95% CI: 0.601–0.852), EB1213 was 0.721 (95% CI: 0.594–0.848), SM (d16:1/20:1) was 0.712 (95% CI): 0.584–0.840), undecylic acid was 0.706 (95% CI: 0.578–0.834), SM (d16:1/16:0) was 0.703 (95%CI: 0.576–0.830), and the AUC of 3alpha-Hydroxy-7-oxo-5alpha-cholan-24-oic Acid was 0.700 (95%CI: 0.571–0.829).

To further explore the potential biomarkers related to AD, a conditional logistic stepwise regression analysis for prenatal depression was performed using the six lipids with the largest AUC values. The results showed that CS (*P* = 0.009) and PC (18:2(2E,4E)/0:0) (*P* = 0.035) were significantly associated with AD (Table 3). As the CS levels in plasma increased, the subjects showed an increased risk of severe AD (OR 1.000125, 95% CI 1.000031–1.000220). Similarly, the risk of severe AD increased with increasing plasma levels of PC (18:2(2E,4E)/0:0) (OR1.000081, 95% CI 1.000006–1.000155).

**Table 3:** Conditional logistic regression for the selection of potential diagnostic biomarkers for AD and NAD pregnant woman

Models	Parameter	b	SE	Waldx^2^	*P*-value
Model 1	Cholesterol sulfate	0.000110	0.000039	7.892	0.005
Model 2	Cholesterol sulfate	0.000125	0.000048	6.768	0.009
	PC(18:2(2E,4E)/0:0)	0.000081	0.000038	4.431	0.035

To investigate whether CS is affected by clinical characteristics of the pregnant women, we performed an additional exploratory multiple regression analysis using the entire model. The results showed that age (*P* = 0.055, approximate statistical significance), marital relationship (*P* = 0.027), and EPDS (*P*<0.001) significantly influenced plasma CS levels (Table 4).

**Table 4:** Multiple regression analysis for the clinical data of antenatal depression women

Variables	Standardized Coefficients	*t* Value	*P* Value
EPDS score	0.506	4.505	< 0.001
Age	0.215	1.956	0.055
BMI	0.123	1.184	0.241
Educational level	−0.047	−0.438	0.663
Marital relationship	−0.258	−2.265	0.027
Sleep quality	0.123	0.273	0.273

Age, body mass index (BMI) and Edinburgh postpartum depression scale (EPDS) were regarded as continuous variables; educational level (9 to 12 years = 1, over 12 years = 2), marital relationship (General = 1, Good = 2) and sleep quality (Very poor – poor = 1, General = 2, Good -very good = 3) were regarded as categorical variables

## Discussion

Many previous studies have attempted to find specific biomarkers for different subtypes of depressive disorders for a more accurate diagnosis. For example, some studies have found that the levels of n-3 polyunsaturated fatty acids (n-3 PUFAs) [29] and mono unsaturated and saturated fatty acids [30] were reduced in adult depressed populations. The expression of PUFAs, capric acid, cis-9-palmitoleic acid, dodecanoic acid, oleic acid, and palmitic acid were found to be reduced in children and adolescents with major depression [31]. Vitamin D [32, 33], BDNF serum levels [34], and regulatory T cell counts [35] have been found to be useful biomarkers for PPD. In addition, some studies have found that an increase in blood and urinary concentrations of 25-hydroxyvitamin D (25(OH) D [36] and 8-isoprostane, respectively [37], was significantly associated with AD. Another study [38] showed that women with untreated AD had higher TNFα levels. Osborne et al. [39] have reported that women with depression during pregnancy had raised inflammatory biomarkers (IL-6, IL-10, TNFα, and vascular endothelial growth factor) and cortisol levels. In addition, Bodnar et al. [40] indicated that some nutritional biomarkers (essential fatty acids, micronutrients, carotenoids) may be a modifiable risk factor for antenatal depression. These previous findings indicated that the biomarkers for different subtypes of depressive disorders were significantly discernible. Although at present, some physiological trends in the depressive state have been discovered, studies investigating potential markers for AD are still lacking.

In recent years, since a clear role of lipids in psychiatric diseases has been established, lipidomic approaches have been used to explore biomarkers for different subtypes of depressive disorders, such as general depression [41], PPD [42], and depression in adolescents and children [31]. Antepartum depression refers to the mood symptoms of the depressive disorder that appear during pregnancy [43]. Because AD and PPD are significantly different in terms of physiological conditions (e.g. hormone levels and maternal and foetal status) and social environment (e.g. working status, confinement, and parent-child relationship), the potential biomarkers of AD and PPD may be different. However, currently there are few studies on biomarkers for AD using lipidomics. In our study, we focused on exploring potential biomarkers for AD using plasma lipidomics. Our results showed that some differentially expressed lipid metabolites were found in the AD group compared with that in the prenatal depression group, including CS and PC (18:2(2E,4E)/0:0). Conditional logistic regression analysis further demonstrated that increased plasma levels of CS and PC (18:2(2E,4E)/0:0) were significantly correlated with an increased risk of severe antenatal depression.

In this study, CS was shown to be the most remarkable differentially expressed metabolite with an AUC of 0.823, and it was significantly higher in the AD group than in the NAD group. Moreover, in the regression analysis, CS was also the most significant risk factor for major AD (*p* = 0.009). CS is an endogenous steroid, and its plasma levels have been demonstrated to modestly but significantly increase during the course of a normal pregnancy [44]. However, the physiologic implication of this interesting finding is presently unclear. To the best of our knowledge, changes in the CS plasma concentrations have not been found in other studies investigating biomarkers for depression. CS can serve as a substrate for the synthesis of pregnenolone sulfate (PregS), [45–48] an endogenous excitatory neurosteroid [49], which is known to improve sleep quality [50, 51] and have antidepressant effects [52, 53]. Therefore, we speculate that in women with AD, synthesis of PregS may be obstructed, thereby inducing increased plasma CS levels.

Through additional multiple regression analyses, we found that marital relationship and EPDS also have an effect on the plasma levels of CS. Age [54] and marital relationship [55] have been demonstrated to be risk factors for AD. Combined with the results that CS plasma levels were positively correlated with AD in this study, these analyses further suggest that plasma CS levels could be affected by high risk factors for AD and this might help us better understand the effects of CS in AD. Based on the current results and previous findings, we speculate that high levels of plasma CS might be an effective and particular predictor of antenatal depression.

Another identified biomarker associated with higher major AD risk was the metabolite PC (18:2(2E,4E)/0:0). It was demonstrated that in the AD group the level of plasma PC was significantly higher than that in the NAD group. This was consistent with another study where increased plasma levels of PC were found in adolescents and children with major depression [31]. In addition, it was recently reported that in patients with bipolar disorder, phosphomonoesters (PC is a kind of phosphomonoester) were higher in the depressive state than that in the euthymic state [56]. Another study, [57, 58] based on magnetic resonance spectroscopy imaging, reported a direct association and showed that PC was higher in depression-related brain regions such as the hippocampus. Therefore, we believe that PC might be another alternative biomarker for AD. According to our literature review, other supporting evidence regarding the biomarkers identified in the current study is not available although some physiological trends in the depressive state have been discovered. Therefore, currently it is difficult to present a comprehensive review on the effects of these identified biomarkers for AD. Based on the current findings, we believe that it is important to further explore the potential mechanisms of these biomarker related to AD.

At present, the EPDS-10 is widely used for screening perinatal depression. However, clinical diagnosis of depression depends on psychiatric interviewing by professionally trained experts. Accurate diagnosis of depression is difficult and time consuming for busy non-psychiatric clinicians, especially for obstetricians and anaesthetists prior to performing a caesarean section. Therefore, it may be helpful to diagnose AD using a process similar to that used for the prediction of gestational trophoblastic diseases (GTD) in routine clinical practice. Obstetricians may be able to identify the possibility of developing GTD based on serum hCG levels with a simple and objective blood test. Similarly, based on the current study, non-psychiatric doctors might easily and objectively predict AD according to the plasma levels of CS. Compared to the EPDS-10, this method may be more convenient and applicable for clinicians who have not undergone psychiatric diagnostic training. However, as noted in the current study, the false-positive rate of the identified biomarker was relatively high. Thus, although analysis of CS plasma levels may be an alternative screening method for the identification of suspected cases, re-assessment by physicians should also be performed for the diagnosis of antenatal depression. Additionally, the sensitivity and specificity of the current biomarkers used for the prediction of AD in clinical practice need further investigation in the future.

### Limitations

Several limitations should be considered when interpreting the current findings. First, the sample size of this study is relatively small; thus, further clinical evidence based on other independent populations is needed to validate our results. Second, in this study, only patients with a full-term pregnancy were recruited 1 day before the caesarean section; thus, whether the current finding may also be applicable to the other trimesters of pregnancy remains unclear. However, two strengths of the current study should be noted. First, the study focused on a specific clinical subcategory of depressed subjects, i.e., antenatal depression, and explored biomarkers associated with major antenatal depression. Female participants with a clinical diagnosis and plasma lipidomics were combined to explore potential biomarkers for antenatal depression. This study may expand the boundaries of the biomarkers of depression and might provide an alternative method for the identification of positively depressed women in the antenatal period. In addition, preoperative preventative screening might help obstetricians to provide a better medical service for women with depression and to enhance their recovery after surgery.

## Conclusion

Women who underwent a caesarean section and experienced antenatal depression presented a significantly different expression profile of plasma lipidic metabolites compared to women who did not experience antenatal depression. CS and PC might be effective and specific lipidic biomarkers for the prediction of antenatal depression. However, further validation studies are needed to confirm the current findings, and the possible detailed mechanism underlying the association between the identified lipidic metabolites and antenatal depression needs to be further studied.

## Data Availability

The study data can be accessed from the corresponding author GD or HL by request.

## Abbreviations

AD: Antenatal depression
AUC: Area under the ROC curve
CS: Cholesterol sulfate
DSM-V: American Psychiatric Association’s Diagnostic and Statistical Manual of Mental Disorders, fifth edition
EPDS-10: 10 Edinburgh postpartum depression
IQR: Interquartile range
NAD: Non-antenatal depression
PCA: Principal component analysis
PLS-DA: Partial least squares method
PregS: Pregnenolone sulfate
ROC: Receiver operating characteristic curve
UPLC-MS: Ultra-performance liquid chromatography-mass spectrometer
VIP: Variable Importance for the Projection
